# Aberrant Structural and Functional Developmental Trajectories in Children With Intellectual Disability

**DOI:** 10.3389/fpsyt.2021.634170

**Published:** 2021-04-13

**Authors:** Xuejin Ma, Jianxia Tan, Lin Jiang, Xuqin Wang, Bochao Cheng, Peng Xie, Yuanyuan Li, Jiaojian Wang, Shiguang Li

**Affiliations:** ^1^Department of Radiology, The First People's Hospital of Zunyi, The Third Affiliated Hospital of Zunyi Medical University, Zunyi, China; ^2^Department of Child Health, The First People's Hospital of Zunyi, The Third Affiliated Hospital of Zunyi Medical University, Zunyi, China; ^3^Department of Radiology, West China Second University Hospital of Sichuan University, Chengdu, China; ^4^Department of Critical Care Medicine, The First People's Hospital of Zunyi, The Third Affiliated Hospital of Zunyi Medical University, Zunyi, China; ^5^School of Life Science and Technology, University of Electronic Science and Technology of China, Chengdu, China; ^6^Center for Language and Brain, Shenzhen Institute of Neuroscience, Shenzhen, China

**Keywords:** intellectual disability, gray matter volume, functional connectivity, development, fronto-parietal network

## Abstract

Intellectual disability (ID) is associated with aberrant structural and functional development of the brain, yet how the dynamical developmental changes of the structure and function of ID from childhood to around puberty remains unknown. To explore the abnormal developmental trajectories of structure and function, 40 children with ID aged 6–13 years and 30 sex-, age-, and educational level-matched healthy controls (HC) with age range from 6 to 13 were recruited. The automatic voxel-based morphometry (VBM) and resting-state functional connectivity (FC) analyses were adopted to delineate the structural and functional differences. Significantly decreased total gray matter volume (GMV) and white matter volume (WMV) in children with ID were found, and the developmental trajectories of GMV and WMV in children with ID showed an opposite direction as compared with HC. The voxel-wise VMB analysis further revealed significantly increased GMV in the dorsal medial prefrontal cortex (dmPFC), bilateral orbital part of the inferior frontal gyrus (orb_IFG.L, orb_IFG.R), right cuneus (cuneus.R), and bilateral middle frontal gyrus (MFG.L, MFG.R) in children with ID. The following seed-based whole-brain functional connectivity analyses of the brain areas with changed GMV found decreased FCs between the cuneus.R and left intraparietal sulcus (IPS.L) and between the MFG.R and anterior cingulate cortex (ACC) in children with ID. Moreover, negative correlations between GMV values in the dmPFC, orb_IFG.L, cuneus.R, and intelligence quotient (IQ) scores and positive correlations between the FCs of the cuneus.R with IPS.L and MFG.R with ACC and IQ scores were found in children with ID and HC. Our findings provide evidence for the abnormal structural and functional development in children with ID and highlight the important role of frontoparietal network in the typical development. The abnormal development of GMV and functional couplings found in this study may be the neuropathological bases of children with ID.

## Introduction

Intellectual disability (ID) is a generalized developmental disorder and is characterized by deficits in both intelligence and social adaptation with intelligence quotient (IQ) scores <70. ID is a global development delay (GDD) disorder which occurs under 5 years of age and accounts for 0.95–3% of children's disability (1–3). ID is strongly associated with environmental and genetic factors, and it is usually accompanied by brain structural and functional abnormalities and other mental disorders (4, 5). Although conventional magnetic resonance imaging (MRI) is able to detect brain aberrant structural alterations like dysplasia of the corpus callosum, enlarged ventricles, and dysplasia of the cerebral cortex closely associated with intellectual disorder (6, 7), a majority of children with ID appear with invisible structural changes on conventional MRI (6, 8). Moreover, whether/how structural changes lead to abnormal functions and behaviors in ID is elusive. Therefore, quantitative structural MRI analyses could contribute toward the biological determination of morphometric changes in ID.

The development of cognitive functions is closely related to the normal processes of neurogenesis, synaptogenesis, and pruning and myelination, and the abnormal developmental trajectories of gray matter and white matter may result in brain functional impairments (9, 10). Through manually measuring the diameter and cross-sectional area, hypoplastic corpus callosum (11) and enlarged supratentorial CSF spaces (12) were identified in children with ID compared with typical development children. Because of manual bias, a fully automatic voxel-based morphometry (VBM) method (13) was used and reduced total brain gray and white matter volume and increased gray matter volume in ACC in children with ID was found (14), but in this study, the demographic information between groups is unmatched and there is a lack of intelligence level on subjects. By analysis of cortical thickness, Zhang and colleagues found reduced cortical thickness in the bilateral lingual gyrus, fusiform gyrus, parahippocampal gyrus, temporal pole, left inferior temporal gyrus, right lateral orbitofrontal cortex, and right precentral gyrus (15). Using diffusion tensor imaging (DTI), degenerated myelination of the uncinate fasciculus, superior cerebellar peduncle, inferior longitudinal fasciculus, corpus callosum, optic radiation, and corticospinal tract and disrupted network topology properties including global and local efficiency and nodal degree have been revealed (16–19). However, all these studies only include either adult or child participants under 5 years of age, and the sample size is also relatively small. Moreover, during development, the period from childhood to around puberty is important for brain functional integration and segregation, high-order cognitive functions, and individual intelligence maturing (20, 21). Therefore, characterizing the structural and functional differences between atypical ID and typical controls from childhood to around puberty could shed new light on the biological and neural bases of ID. To delineate the structural and functional abnormalities in atypical development ID participants, the voxel-wise VBM and resting-state functional connectivity analyses were adopted in this study to identify the aberrant developmental trajectories of brain structures and functions from childhood to around puberty/early adolescence.

## Materials and Methods

### Participants

Forty children with ID (26 males/14 females, age: 9.70 ± 2.07 years) diagnosed based on the Diagnostic and Statistical Manual of Mental Disorders (DSM-V) (3) were recruited from the First People's Hospital of Zunyi (from December 2018 to January 2020). Thirty age-, sex-, and education-matched healthy controls (HC, 18 males/12 females, age: 9.51 ± 1.54 years) were also recruited. All participants received MRI examination and intelligent assessment using the Manual for the Wechsler Intelligence Scale for Children-Revised (WISC-R) (22). The inclusion criteria for ID were as follows: (a) deficit in both intelligence and adaptive functions such as reasoning, problem-solving, planning, abstract thinking, academic learning, and social communication and onset during the developmental period; (b) intelligence quotient (IQ) scores <70; (c) no cause of secondary ID, such as tumor, trauma, and tuberous sclerosis in the brain; (d) right-handedness; and (e) aged 6–13 years. The exclusion criteria of ID were as follows: (a) had a contraindication for MRI; (b) fell asleep during rs-fMRI scanning; (c) had a history of neurological disorders other than ID; (d) had focal abnormality with conventional MRI like dysplasia of the corpus callosum, enlarged ventricles, and posterior fossa subarachnoid space; and (e) had abnormal height and weight based on WHO Growth reference data for 5–19 years (23). In the HC group, all the children are right-handed and had IQ scores >90. The written informed consents were given and obtained from all the participants or their guardians. This study was approved by the local ethics committee of the First People's Hospital of Zunyi.

### MRI Data Acquisition

MRI data were acquired with a SIEMENS 3.0-T (Magnetom Skyra, Siemens-Healthcare) scanner using a standard head coil at the Department of Radiology, The First People's Hospital of Zunyi. All the subjects were instructed to stay at rest with their eyes closed but keep awake and not think of anything. For each subject, resting-state functional images were acquired using a gradient-recalled echo-planar imaging (EPI) sequence with the following parameters: repetition time = 2,000 ms, echo time = 30 ms, flip angle = 90°, 30 axial slices covering full brain, field of view = 240 × 240 mm^2^, slice thickness = 5 mm with no gap, voxel size = 3 × 3 × 5 mm^3^, and 210 volumes were obtained. Three-dimensional T1-weighted imaging (3D-T1WI) images were also acquired by using a 3D-T1WI (magnetization prepared rapid gradient echo, MPRAGE) sequence with parameters of field of view = 230 × 230 mm^2^, repetition time = 2,200 ms, echo time = 2.48 ms, inversion time = 900 ms, flip angle = 8°, and slice thickness = 1.00 mm, covering 192 axial slices with an in-plane resolution of 0.9 mm × 0.9 mm.

### Intelligent Assessments

The participants meeting our inclusion criteria of MRI and physical examinations needed to take an intelligent test (WISC-R). The tests were performed by one of our colleagues (X.W) who is qualified for clinical children's neuropsychological evaluation. Tests were completed in one quiet room at the Department of Child Health of the First People's Hospital of Zunyi with a professional toolbox. The whole process included items of information, comprehension, arithmetic, similarities, vocabulary, digit span, picture completion, picture arrangement, block design, object assembly, coding, and mazes. According to the manual of WASI-II (Chinese version), during each item, the tester asked questions or gave instructions to the child and scored the child's answers or performance, and finally calculated the intelligence quotient score of the child.

### VBM Analysis

High-spatial-resolution T1-weighted MRI data were processed with VBM Toolbox (VBM8; http://dbm.neuro.uni-jena.de/wordpress/vbm/) in statistical parametric mapping software (SPM8; http://www.fil.ion.ucl.ac.uk/spm/software/spm8/). First, all images with artifacts were removed. Second, according to default settings in VBM8, images were segmented into gray matter (GM), white matter (WM), and cerebrospinal (CSF) areas and then normalized to Montreal Neurologic Institute (MNI) space and modulated. After checking the segmentation quality, the segmented GM images were smoothed by using an 8-mm full width at half-maximum Gaussian kernel for subsequent group comparisons. The independent two-sample *t*-test with the total brain volume as covariate was used to identify the significant differences in GMV between ID and HC groups. The significant level was determined using the false discovery rate (FDR) method with *p* < 0.001 and minimum cluster size >100 voxels.

### Resting-State Functional Connectivity Analysis

The preprocessing of functional images was performed using the Data Processing Assistant for Resting-State fMRI (DPARSF) (24) and statistical parametric mapping (SPM8, http://www.fil.ion.ucl.ac.uk/spm/software/spm8/). To avoid instability of the magnetic field, the first 10 volumes were removed, and the remained volumes were realigned to the first volume to correct the head motion. After realigning, the functional images were normalized to a standard EPI template in the MNI space and resampled to a voxel resolution of 3 × 3 × 3 mm^3^. Next, all the volumes were smoothed with 8-mm FWHM Gaussian kernels and detrended. The nuisance covariates containing Friston 24-parameter head motion estimates, averaged time series in white matter, and cerebrospinal fluid were then regressed. Finally, all the fMRI images were filtered with a temporal band path of 0.01–0.1 Hz. To exclude the head motion effects, the subjects were excluded if the head motion exceeds 3 mm of translation or 3° of rotation. Under this criterion, 3 subjects in the ID group and 3 subjects in the HC group were excluded. Moreover, we calculated the frame-wise displacement (FD) value for each volume and censored the bad images (before 2 volumes and after 1 volume) with FD >0.5 (25). The global signal was not regressed to avoid introducing false-negative correlations and eliminate positive correlations (26, 27).

Seed-based whole-brain FC analysis was used to explore the functional differences between ID and HC groups. To calculate FC, the seed areas were first defined by creating spheres with a 6-mm radius based on the peak coordinates yielded by aforementioned group comparisons of GMV. Next, the mean time series of each seed area was calculated, and the Pearson correlation coefficients between the time series of each seed area and the remaining voxels of the rest of the brain were calculated and converted to z scores by Fisher z-transformation. Then, a voxel-wise whole-brain FC map was obtained for each subject. Finally, independent two-sample *t*-tests were used to identify the FC differences in children with ID. The significant level was determined using cluster-level Alphasim correction method with *p* < 0.05 (cluster-forming threshold at voxel-level *p* < 0.001).

### Correlation Analysis

Correlation coefficients between IQ scores and the GMV and FC values in brain areas with significant differences between ID and HC groups were performed in all subjects, and the significant level was set at *p* < 0.05 with Bonferroni correction.

## Results

### Demographics and Clinical Characteristics

Table 1 shows the detailed demographics and clinical characteristics for the used subjects in this study. There were no significant differences in age (*p* = 0.67), education level (*p* = 0.87), and sex (*p* = 0.67) between the ID and HC groups (Table 1). No significant difference in head motion of FD values (*p* = 0.30) was observed between the two groups (Table 1).

**Table 1:** Characteristics of ID and HC.

**Characteristics**	**ID (*n* = 40)**	**HC (*n* = 30)**	
	**Mean ± SD**	**Mean ± SD**	***P*-value**
Sex (male/female)	26/14	18/12	0.67^‡^
Age, years	9.70 ± 2.07	9.51 ± 1.54	0.67*^†^*
Handedness (right/left)	40/0	30/0	–
Education, years	3.43 ± 1.77	3.35 ± 1.70	0.87*^†^*
IQ scores	56.00 ± 7.68	107.20 ± 9.81	<0.0001*^†^*
FD power	0.28 ± 0.35	0.20 ± 0.14	0.30*^†^*

*ID, intellectual disability; HC, healthy control; n, number of subjects; SD, standard deviation; IQ, intelligence quotient; FD, framewise displacement*.

‡ *Chi-squared test was used*;

† *Two sample t-test was used*.

### Between-Group Comparisons of GMV and WMV

**Figure 1:**
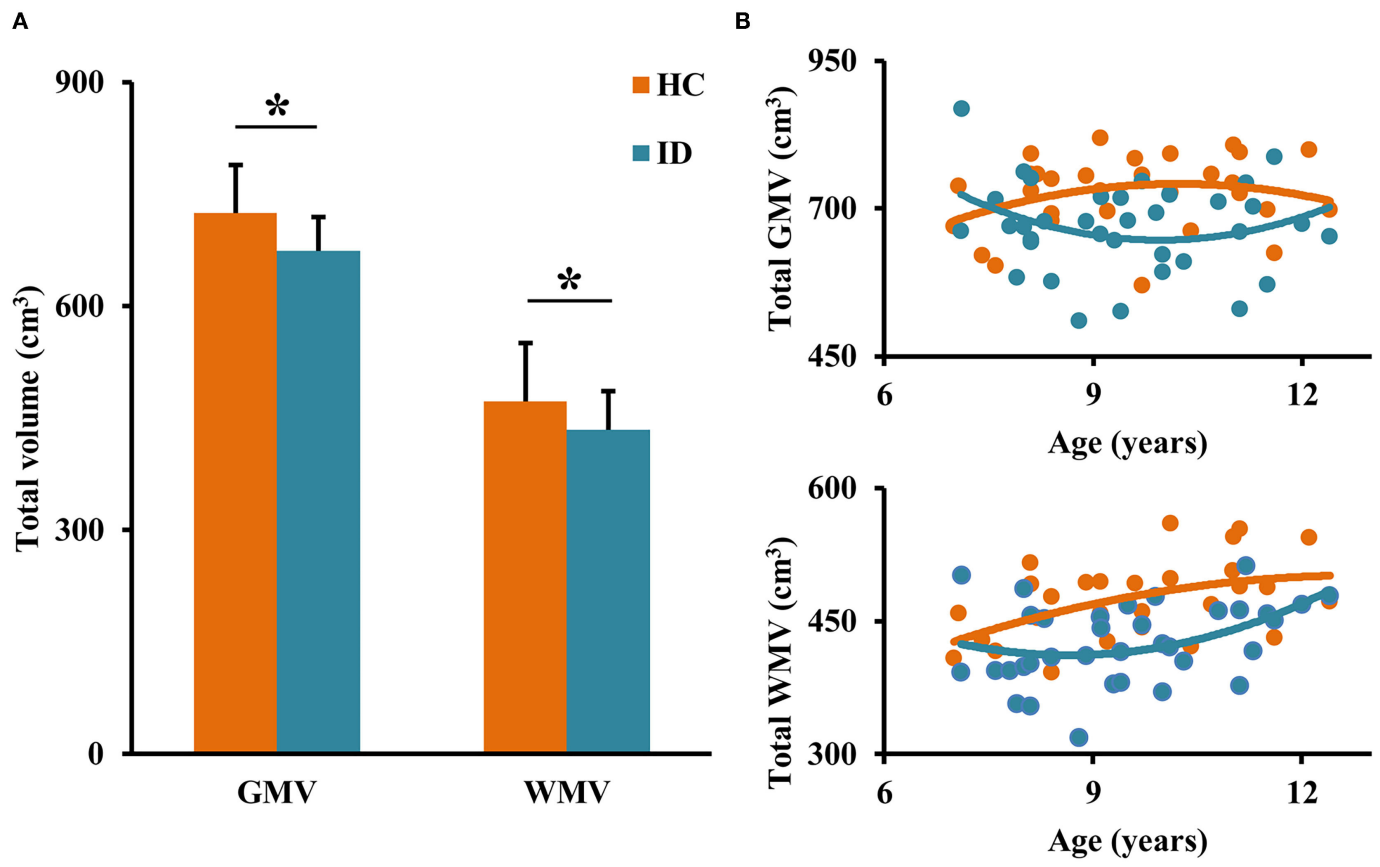
Between-group comparisons of total gray matter volume (GMV) and white matter volume (WMV). **(A)** Decreased total GMV and WMV were detected in children with ID compared to healthy controls (HC). **(B)** Different developmental trajectories of the total brain GMV and WMV were found in ID and HC. *represents significant differences.

First, we compared the total brain GMV and WMV to determine whether ID disorder results in global developmental delay. Between-group comparisons of total GMV and WMV found significantly decreased total GMV (*p* = 0.0049) and WMV (*p* = 0.0020) in children with ID (Figure 1). The developmental trajectories of GMV and WMV in healthy controls are U-shaped, whereas the developmental trajectories of GMV and WMV in children with ID are inverted U-shaped (Figure 1).

**Figure 2:**
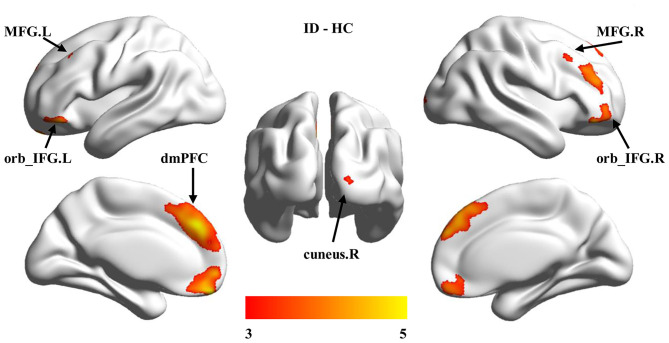
Group differences in gray matter volume (GMV). Voxel-based morphology (VBM) was used to identify voxel-wise changes of GMV in ID participants. Significantly increased GMV was found in the dorsal medial prefrontal cortex (dmPFC), bilateral orbital part of the inferior frontal gyrus (orb_IFG.L, orb_IFG.R), right cuneus (cuneus.R), and bilateral middle frontal gyrus (MFG.L, MFG.R) in ID compared to HC.

To further determine the specific brain areas with changed GMV, the voxel-wise between-group comparisons of GMV were performed. Significantly increased GMVs in the dorsal medial prefrontal cortex (dmPFC), the bilateral orbital part of inferior frontal gyrus (orb_IFG.L, orb_IFG.R), the bilateral middle frontal gyrus (MFG.L, MFG.R), and the right cuneus (cuneus.R) were found in ID compared to HC (Figure 2, Table 2).

**Table 2:** Brain regions showing increased GMV in ID vs. HC.

**Brain regions**	**Peak MNI coordinates**			**Peak *t*-value**	**Cluster size**
	**X**	**Y**	**Z**		
dmPFC	−9	51	−19.5	5.2083	8069
orb_IFG.L	−51	36	−16.5	5.1772	983
orb_IFG.R	54	36	13.5	4.7254	3588
cuneus	6	−99	18	4.2991	419
MFG.L	−31.5	12	45	4.561	261
MFG.R	46.5	10.5	54	4.2053	129

*GMV, gray matter volume; ID, intelligent disability; HC, healthy control; dmPFC, dorsal medial prefrontal cortex; orb_IFG, orbital inferior frontal gyrus; MFG, middle frontal gyrus*.

### Between-Group Comparisons of Seed-Based FCs

**Figure 3:**
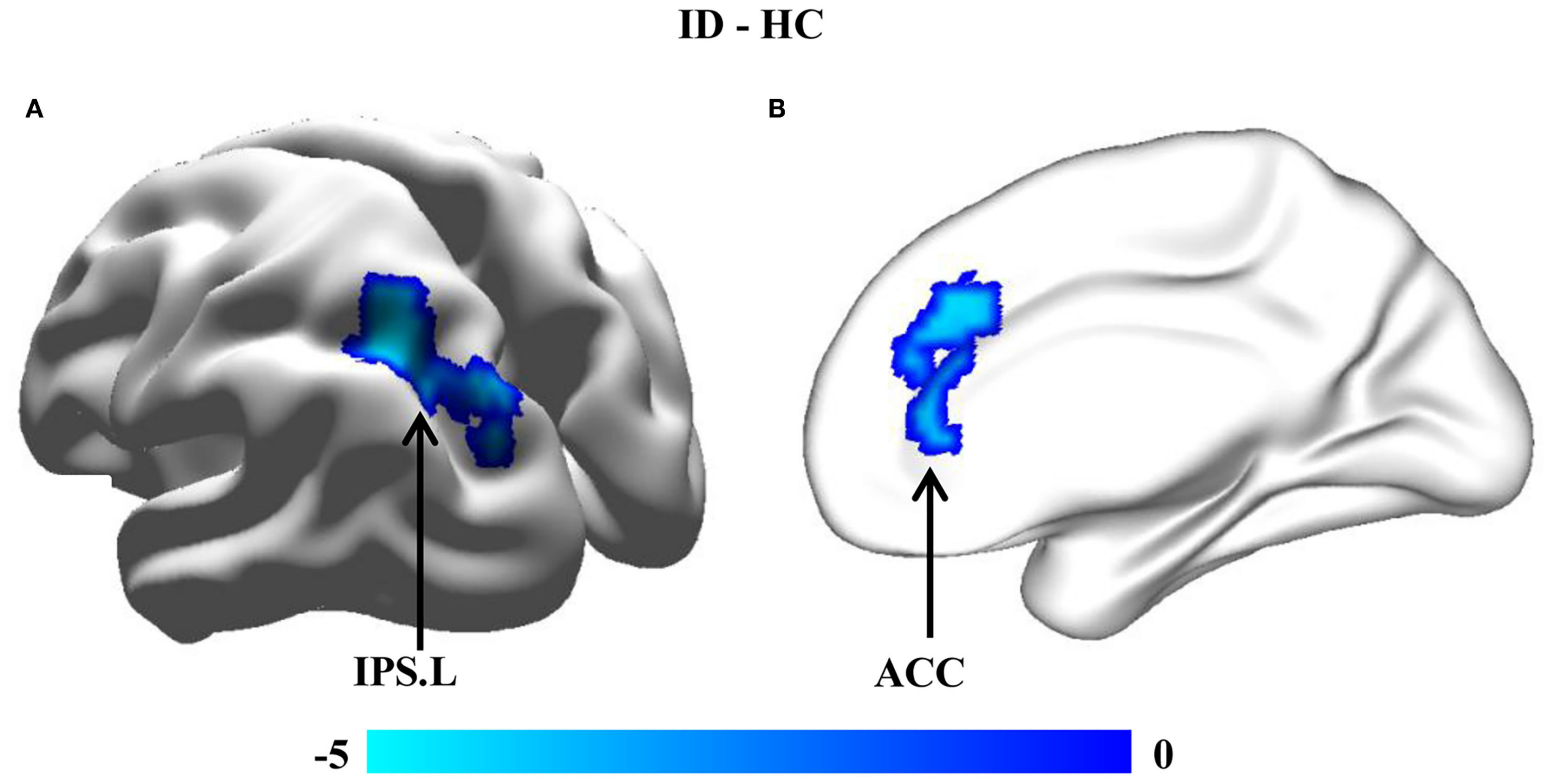
Between-group comparisons of resting-state functional connectivity (FC). Seed-based FC analyses of brain areas showing that changed GMVs were performed to reveal the abnormal functional couplings. Significantly decreased FCs **(A)** between the cuneus.R and left intraparietal sulcus (IPS.L) and **(B)** between the MFG.R and anterior cingulate cortex (ACC) were found in individuals with ID compared to HC.

Whole-brain voxel-wise FC analyses found significantly reduced FC between the cuneus.R and left intraparietal sulcus (IPS.L) [peak *t*-value = −4.61 and MNI coordinates = [−30, −57, 45]] (Figure 3A) and between the MFG.R and anterior cingulate cortex (ACC) [peak *t*-value = −4.26 and MNI coordinates = [15, 30, 39]] in children with ID compared to HC (Figure 3B).

### Brain–Behavior Associations

**Figure 4:**
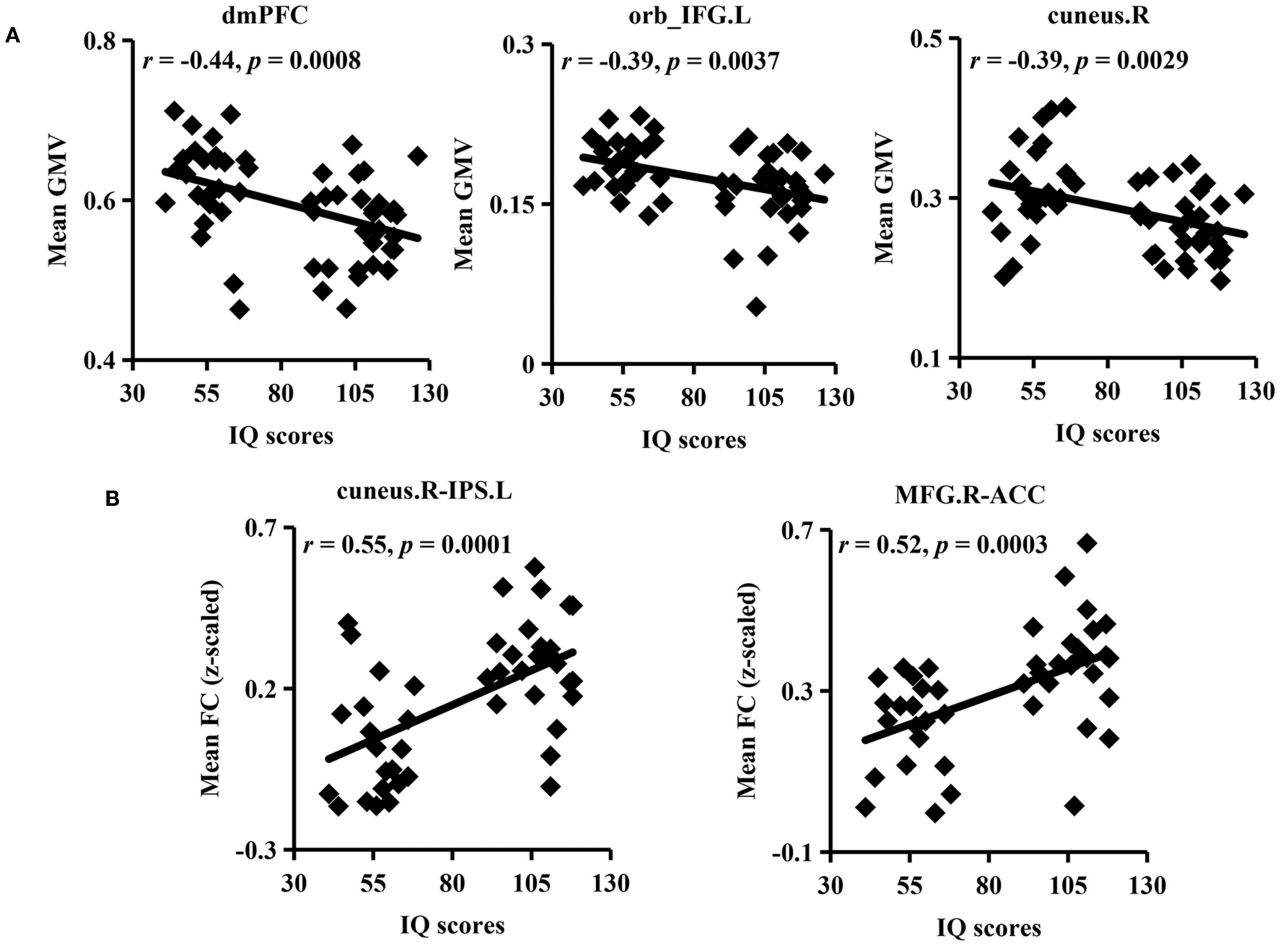
Association analyses. **(A)** Significantly negative correlations were found between intelligence quotient (IQ) scores and mean GMV in the dmPFC, orb_IFG.L, and cuneus.R in ID and HC participants. **(B)** Mean FCs between the cuneus.R and IPS.L and between the MFG.R and ACC were significantly positively correlated with IQ scores.

To explore the relationship between IQ and GMV, FC, correlation analyses were further applied (Figure 4). Significantly negative associations were found between mean GMV of the dmPFC, orb_IFG.L, cuneus.R, and IQ scores. Meanwhile, FCs between the cuneus.R and IPS.L and between the MFG.R and ACC were positively correlated with IQ scores.

## Discussion

The voxel-based morphometry and FC analyses were employed to investigate the structural and functional developmental abnormalities in children with ID from childhood to around puberty. Children with ID showed decreased global GMV and WMV while increased frontal and occipital GMV. The resting-state FC analyses further revealed decreased functional couplings in attention and executive control networks in ID. The presence of specific structural and functional alterations indicated that children with ID have abnormal developmental trajectory from childhood to around puberty, which may be the neural basis of functional disorders observed in ID.

### Altered Maturational Patterns

Our results of significantly decreased total volume of GMV and WMV in ID compared to HC are in accordance with previous findings (14, 20, 28–30). The higher GMV and WMV in HC are mainly related to a larger number of neuron cells and fibers for better computational and communicational ability (31). Although most mature processes of the brain are completed before age 2 years like shape, volume, and fold, and the brain size reaches about 90% of adult size by 5 years of age, it keeps plasticity across the lifespan (32). In our study, we found that the total GMV in HC increases to a peak (about 10–11 years) and then decreases while the WMV keeps increasing. The development trajectories of GMV and WMV in typical children is in line with that found in a previous study (32). During development, the increasing GMV and WMV mainly come from continuous generation of dendrite, axon, and synapse and myelination while decreasing GMV and WMV may result from competitive elimination of redundant synapse. This process serves as the neural substrate of development of the primary system and high-order cognitive functions (10). In the ID group in our study, the developmental courses of total GMV and WMV showed almost opposite directions compared to that in HC, which suggests that delayed development of gray and white matters may be the neuropathology of children with ID.

Different from decreases in global GMV and WMV compared with HC, increased region-specific GMV in the prefrontal and occipital cortices were found in ID. Accumulating evidence supports that the frontal cortex is important to both primary and high-order cognition functions. For example, the prefrontal cortex (PFC) has been demonstrated to be involved in memory (32), cognitive control, decision-making (33), language (34, 35), and reasoning (36), and the volume of the PFC is closely related to intelligence level (15, 20, 21). Moreover, the increased GMV in ACC is also supported by a previous study of children with ID (12). Additionally, Zhang and colleagues also found decreased cortical thickness in the right lateral orbitofrontal cortex in adults with ID (15). In a word, increased GMV at the prefrontal regions and ACC in children with ID found in our study may be related to the compensatory effect of hypofunctions of high-order cognitive abilities (10, 21).

### Altered Functional Couplings in the Attention Network

The dorsal attention network (DAN), which mainly comprises the frontal eye fields (FEF), IPS, and the superior occipital gyrus, is involved in goal-directed attention processes and is associated with working memory and intelligence (34, 37, 38). Moreover, the DAN plays a key role in top-down attention control to guide individuals to filter irrelevant information for better task performance. In our study, we found decreased FC between the IPS.L and cuneus.R, two important nodes of the DAN suggesting impaired attention ability and declined cognitive functions in individuals with ID (39). Our findings further highlight the important role of the DAN in intelligence.

In addition to the top-down control of attention by the DAN, the attention-related process also contains bottom-top competitive stimulation selection by the ventral attention network (VAN), which is strongly associated with social cognition (39). Besides, the MFG is regarded as a converter between the DAN and VAN by sending signals from the DAN to direct VAN focusing on the targets and receiving feedback and environmental information from the VAN to DAN for reorienting attention (37). After receiving the important or interesting target information from the external attention system, the salience network, mainly composed of the ACC and the anterior insula, is enrolled to identify most-concerned or nonsense stimulation for dynamic switching between the default mode network and central executive network to regulate the following behaviors (40, 41). Thus, the decreased FCs within the attention network and between the ACC and MFG indicated relatively lower dynamic interaction within the attention network and lower dynamic modulation by the ACC and MFG in children with ID compared to typically developmental children, which may be the underlying basis of the manifested low intelligence level.

There are several limitations in our study. First, the scanning slice is a little thick, which may contribute to inhomogeneity with the voxel and lead to missing subtle but important findings. Second, the sample size of our study is relatively small, and the findings should be validated in large samples in future studies.

## Conclusion

Sustaining plasticity of the brain structure and function is crucial for intellectual development. Apart from aberrant gray and white matter volume alterations, decreased functional connections within the attention system and between attention and the execution system was uncovered. Our findings provide biological and neural basis for children with ID and may guide future interventions.

## Data Availability Statement

The raw data supporting the conclusions of this article will be made available by the authors, without undue reservation.

## Ethics Statement

The studies involving human participants were reviewed and approved by the First People's Hospital of Zunyi. Written informed consent to participate in this study was provided by the participants' legal guardian/next of kin.

## Author Contributions

SL and JW contributed to the conception and design, conducted the data analysis, and drafted and approved the final manuscript. XM contributed to the data analysis, drafting, and revision of the manuscript. XW, JT, and YL offered data collection. SL, LJ, and BC contributed to radiological expertise and helped to select and assess cases. PX provided a critical revision of the manuscript for important intellectual content. All authors have read and approved the final manuscript.

## Conflict of Interest

The authors declare that the research was conducted in the absence of any commercial or financial relationships that could be construed as a potential conflict of interest.

## References

[1] Maulik PK Mascarenhas MN Mathers CD Dua T Saxena S. Prevalence of intellectual disability: a meta-analysis of population-based studies. Res Dev Disabil. (2011) 32:419–36. 10.1016/j.ridd.2010.12.018 21236634

[2] Xie ZH Bo SY Zhang XT Liu M Zhang ZX Yang XL. Sampling survey on intellectual disability in 0 approximately 6-year-old children in China. J Intellect Disabil Res. (2008) 52:1029–38. 10.1111/j.1365-2788.2008.01048.x 18565130

[3] American-Psychiatric-Association. Diagnostic and Statistical Manual of Mental Disorders. 5th ed. American Psychiatric Association, Arlington, VA (2013).

[4] Amor DJ. Investigating the child with intellectual disability. J Paediatr Child Health. (2018) 54:1154–8. 10.1111/jpc.14202 30294993

[5] Hamdan FF Gauthier J Spiegelman D Noreau A Yang Y Pellerin S. Mutations in SYNGAP1 in autosomal nonsyndromic mental retardation. N Engl J Med. (2009) 360:599–605. 10.1056/NEJMoa0805392 19196676 PMC2925262

[6] Rocha AFD Leite CDC Rocha FT Massad E Cerri GG Angelotti SADO. Mental retadation: a MRI study of 146 Brazilian children. Arq Neuropsiquiatr. (2006) 62:186–92. 10.1590/S0004-282X2006000200003 16791353

[7] Habibullah H Albaradie R Bashir S. MRI evaluation of global developmental delay: a retrospective study. Dubai Med J. (2020) 3:1–4. 10.1159/000506900

[8] Murias K Moir A Myers KA Liu I Wei XC. Systematic review of MRI findings in children with developmental delay or cognitive impairment. Brain Dev. (2017) 39:644–55. 10.1016/j.braindev.2017.04.006 28457518

[9] Dennis M Spiegler BJ Juranek JJ Bigler ED Snead OC Fletcher JM. Age, plasticity, and homeostasis in childhood brain disorders. Neurosci Biobehav Rev. (2013) 37:2760–73. 10.1016/j.neubiorev.2013.09.010 24096190 PMC3859812

[10] Ismail FY Fatemi A Johnston MV. Cerebral plasticity: windows of opportunity in the developing brain. Eur J Paediatr Neurol. (2017) 21:23–48. 10.1016/j.ejpn.2016.07.007 27567276

[11] Erbetta A Bulgheroni S Contarino VE Chiapparini L Esposito S Annunziata S. Low-functioning autism and nonsyndromic intellectual disability. J Child Neurol. (2015) 30:1658–63. 10.1177/0883073815578523 25895913

[12] Mannerkoski M Heiskala H Raininko R Åberg L Sarna S Wirtavuori K. Brain magnetic resonance imaging of siblings from families with two or more children with learning or intellectual disabilities and need for full-time special education. Acta Radiol. (2009) 50:437–45. 10.1080/02841850902756524 19267273

[13] Ashburner J Friston KJ. Voxel-based morphometry–the methods. Neuroimage. (2000) 11:805–21. 10.1006/nimg.2000.0582 10860804

[14] Mannerkoski MK Heiskala HJ Leemput KV Åberg LE Raininko R Hämäläinen J. Subjects with intellectual disability and familial need for full-time special education show regional brain alterations: a voxel-based morphometry study. Pediatr Res. (2009) 66:306–11. 10.1203/PDR.0b013e3181b1bd6a 19531975

[15] Zhang Y Wu Y Zhu M Wang C Wang J Zhang Y. Reduced cortical thickness in mental retardation. PLoS ONE. (2011) 6:e29673. 10.1371/journal.pone.0029673 22216343 PMC3246471

[16] Jeong JW Sundaram S Behen ME Chugani HT. Differentiation of speech delay and global developmental delay in children using DTI tractography-based connectome. AJNR Am J Neuroradiol. (2016) 37:1170–7. 10.3174/ajnr.A4662 26797142 PMC4907870

[17] Ramli N Yap A Muridan R Seow P Rahmat K Fong CY. Microstructural abnormalities found in uncinate fasciculus and superior cerebellar tracts in children with global developmental delay: a feasibility study. Clin Radiol. (2020) 75:77 e15–22. 10.1016/j.crad.2019.09.134 31668796

[18] Sundaram SK Sivaswamy L Makki MI Behen ME Chugani HT. Absence of arcuate fasciculus in children with global developmental delay of unknown etiology: a diffusion tensor imaging study. J Pediatr. (2008) 152:250–5. 10.1016/j.jpeds.2007.06.037 18206698

[19] Yu C Li J Liu Y Qin W Li Y Shu N. White matter tract integrity and intelligence in patients with mental retardation and healthy adults. Neuroimage. (2008) 40:1533–41. 10.1016/j.neuroimage.2008.01.063 18353685

[20] Reiss AL Abrams MT Singer HS Ross JL Denckla MB. *Brain* development, gender and IQ in children: a volumetric imaging study. Brain. (1996) 119:1763–74. 10.1093/brain/119.5.1763 8931596

[21] Shaw P Greenstein D Lerch J Clasen L Lenroot R Gogtay N. Intellectual ability and cortical development in children and adolescents. Nature. (2006) 440:676–9. 10.1038/nature04513 16572172

[22] Wechsler D. Manual for the Wechsler Intelligence Scale for Children-Revised. New York, NY: Psychological Corporation (1974).

[23] World-Health-Organization. WHO Growth Reference Data for 5-19 Years. Geneva: World Health Organization (2007). Available online at: www.who.int/growthref/en/

[24] Yan CG Zang YF. DPARSF: A MATLAB toolbox for “Pipeline” data analysis of resting-state fMRI. Front Syst Neurosci. (2010) 4:13. 10.3389/fnsys.2010.00013 20577591 PMC2889691

[25] Power JD Barnes KA Snyder AZ Schlaggar BL Petersen SE. Spurious but systematic correlations in functional connectivity MRI networks arise from subject motion. Neuroimage. (2012) 59:2142–54. 10.1016/j.neuroimage.2011.10.018 22019881 PMC3254728

[26] Gee DG Biswal BB Kelly C Stark DE Margulies DS Shehzad Z. Low frequency fluctuations reveal integrated and segregated processing among the cerebral hemispheres. Neuroimage. (2011) 54:517–27. 10.1016/j.neuroimage.2010.05.073 20570737 PMC3134281

[27] Murphy K Birn RM Handwerker DA Jones TB Bandettini PA. The impact of global signal regression on resting state correlations: are anti-correlated networks introduced? Neuroimage. (2009) 44:893–905. 10.1016/j.neuroimage.2008.09.036 18976716 PMC2750906

[28] McDaniel M. Big-brained people are smarter: a meta-analysis of the relationship between *in vivo* brain volume and intelligence. Intelligence. (2005) 33:337–46. 10.1016/j.intell.2004.11.005

[29] Nave G Jung WH Karlsson Linner R Kable JW Koellinger PD. Are bigger brains smarter? Evidence from a large-scale preregistered study. Psychol Sci. (2019) 30:43–54. 10.1177/0956797618808470 30499747

[30] Spencer MD Moorhead TW Lymer GK Job DE Muir WJ Hoare P. Structural correlates of intellectual impairment and autistic features in adolescents. Neuroimage. (2006) 33:1136–44. 10.1016/j.neuroimage.2006.08.011 16996749

[31] Hilger K Winter NR Leenings R Sassenhagen J Hahn T Basten U. Predicting intelligence from brain gray matter volume. Brain Struct Function. (2020) 225:2111–29. 10.1007/s00429-020-02113-7 32696074 PMC7473979

[32] Lenroot RK Giedd JN. Brain development in children and adolescents: insights from anatomical magnetic resonance imaging. Neurosci Biobehav Rev. (2006) 30:718–29. 10.1016/j.neubiorev.2006.06.001 16887188

[33] Santarnecchi E Emmendorfer A Pascual-Leone A. Dissecting the parieto-frontal correlates of fluid intelligence: a comprehensive ALE meta-analysis study. Intelligence. (2017) 63:9–28. 10.1016/j.intell.2017.04.008

[34] Torre GA Matejko AA Eden GF. The relationship between brain structure and proficiency in reading and mathematics in children, adolescents, and emerging adults. Dev Cogn Neurosci. (2020) 45:100856. 10.1016/j.dcn.2020.100856 32949854 PMC7502824

[35] Wang J Yang Y Zhao X Zuo Z Tan L-H. Evolutional and developmental anatomical architecture of the left inferior frontal gyrus. NeuroImage. (2020) 222:117268. 10.1016/j.neuroimage.2020.117268 32818615

[36] Vincent JL Kahn I Snyder AZ Raichle ME Buckner RL. Evidence for a frontoparietal control system revealed by intrinsic functional connectivity. J Neurophysiol. (2008) 100:3328–42. 10.1152/jn.90355.2008 18799601 PMC2604839

[37] Corbetta M Patel G Shulman GL. The reorienting system of the human brain: from environment to theory of mind. Neuron. (2008) 58:306–24. 10.1016/j.neuron.2008.04.017 18466742 PMC2441869

[38] Lei X Wang Y Yuan H Mantini D. Neuronal oscillations and functional interactions between resting state networks. Hum Brain Mapp. (2014) 35:3517–28. 10.1002/hbm.22418 25050432 PMC6869195

[39] Corbetta M Shulman GL. Control of goal-directed and stimulus-driven attention in the brain. Nat Rev Neurosci. (2002) 3:201–15. 10.1038/nrn755 11994752

[40] Menon V Uddin LQ. Saliency, switching, attention and control: a network model of insula function. Brain Struct Funct. (2010) 214:655–67. 10.1007/s00429-010-0262-0 20512370 PMC2899886

[41] Uddin LQ. Salience processing and insular cortical function and dysfunction. Nat Rev Neurosci. (2015) 16:55–61. 10.1038/nrn3857 25406711

